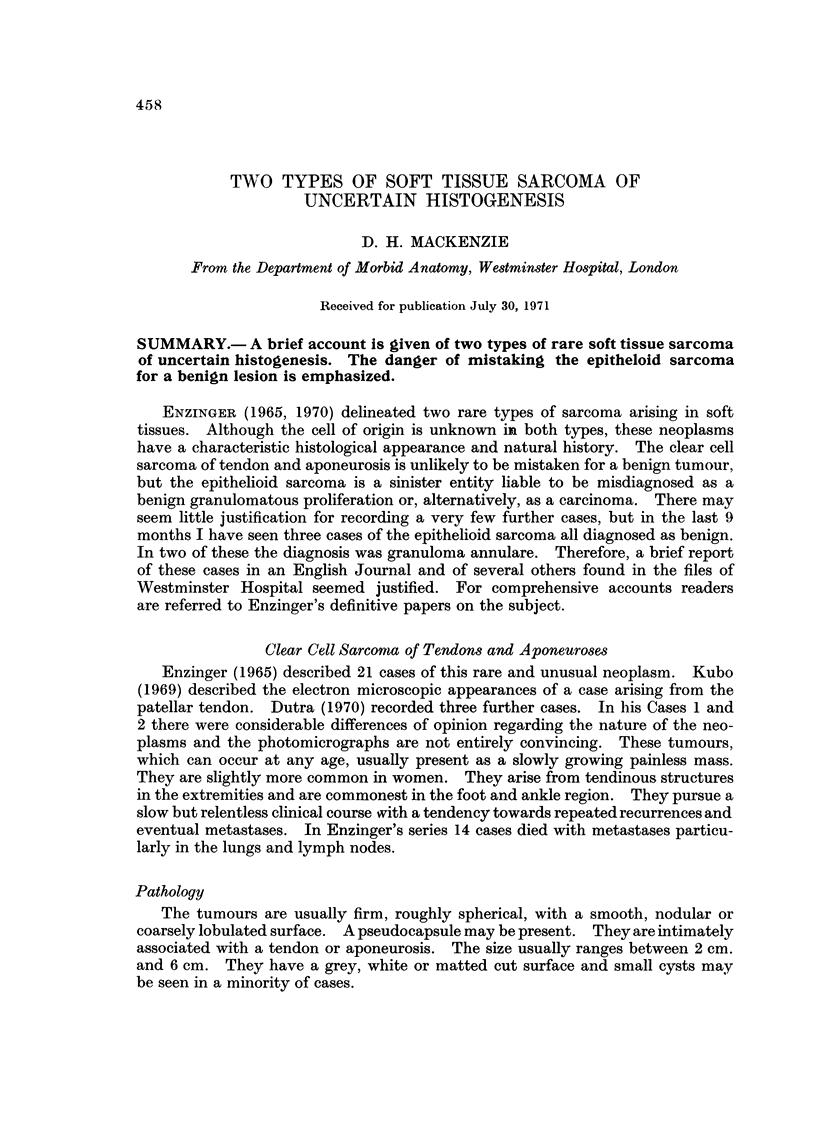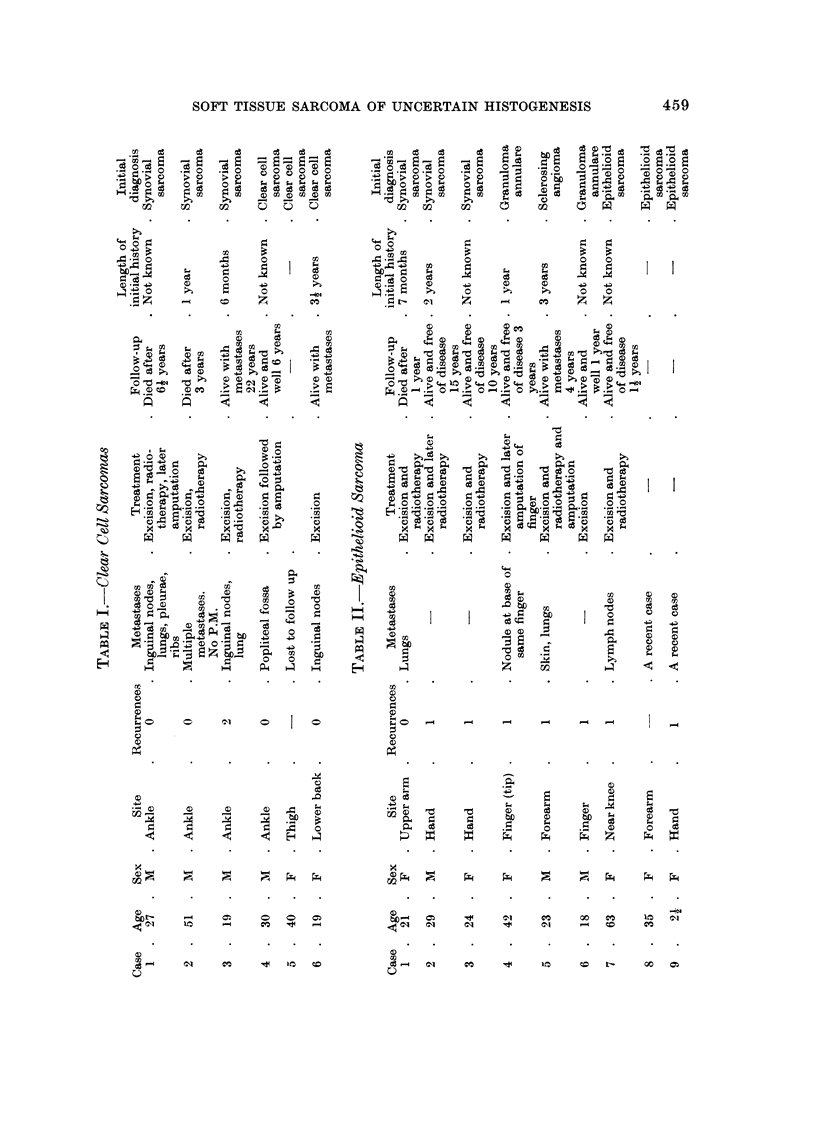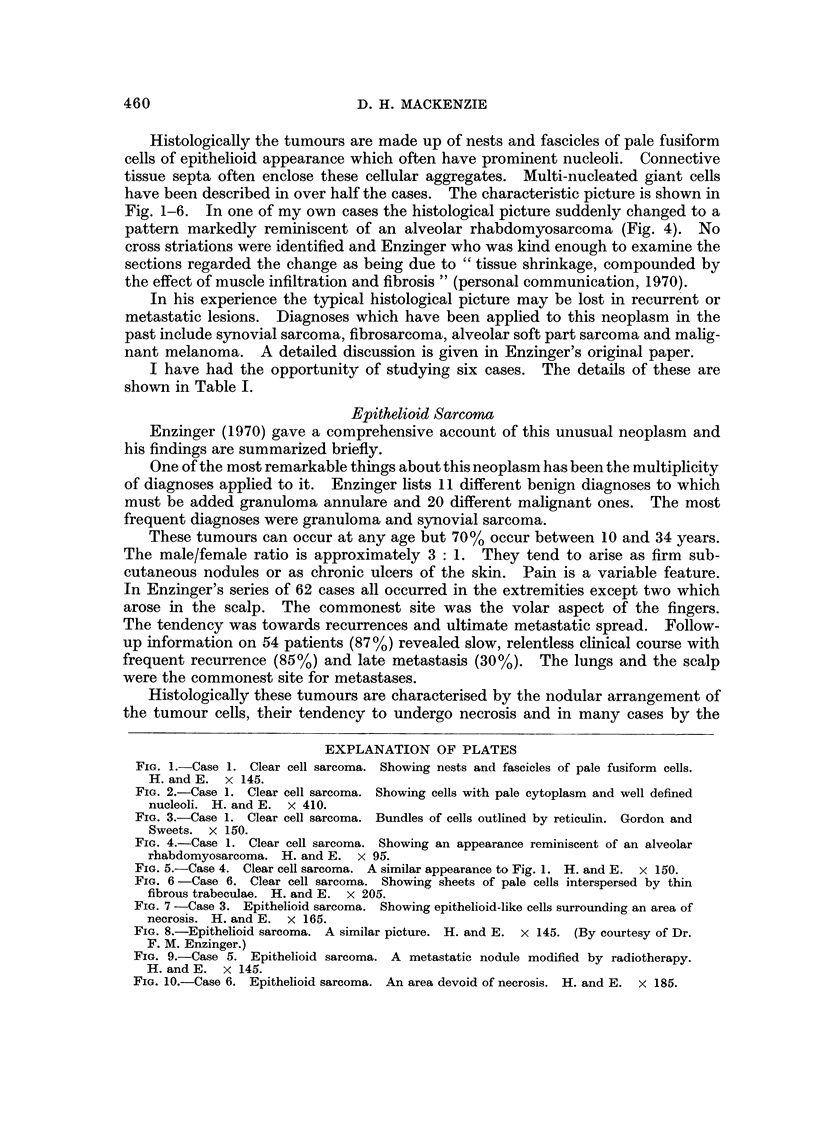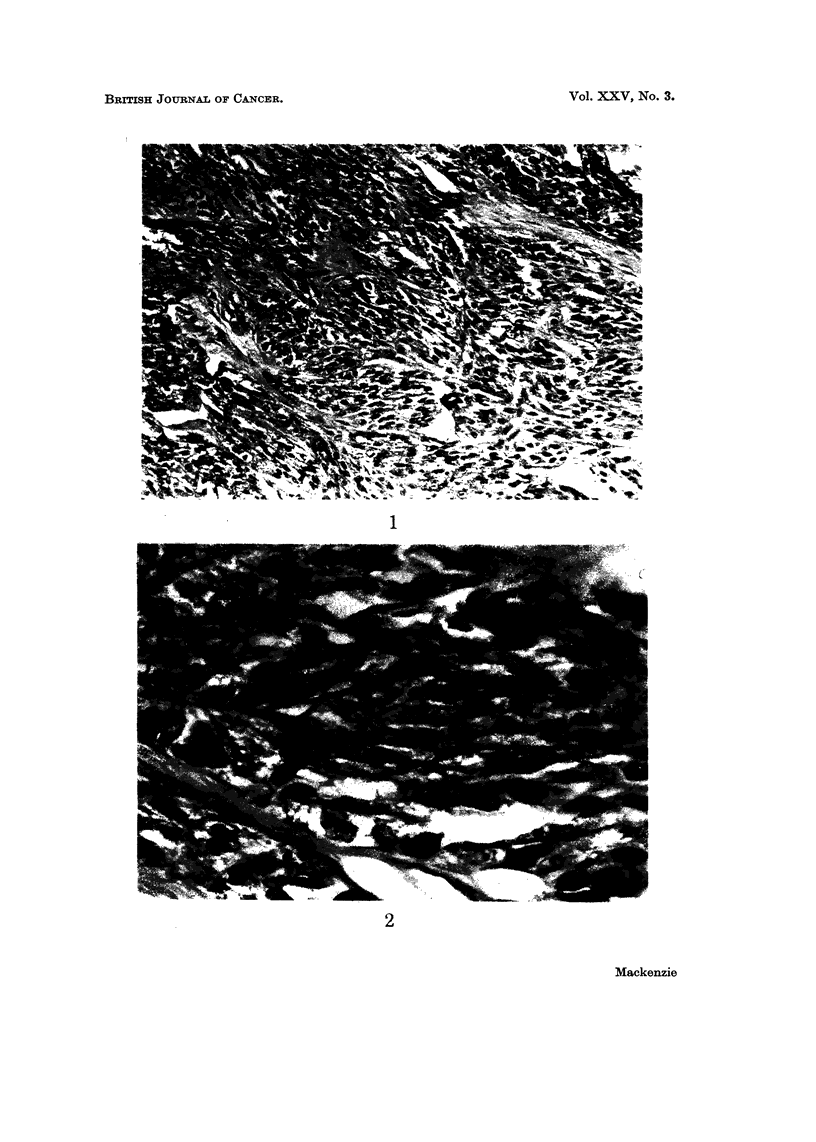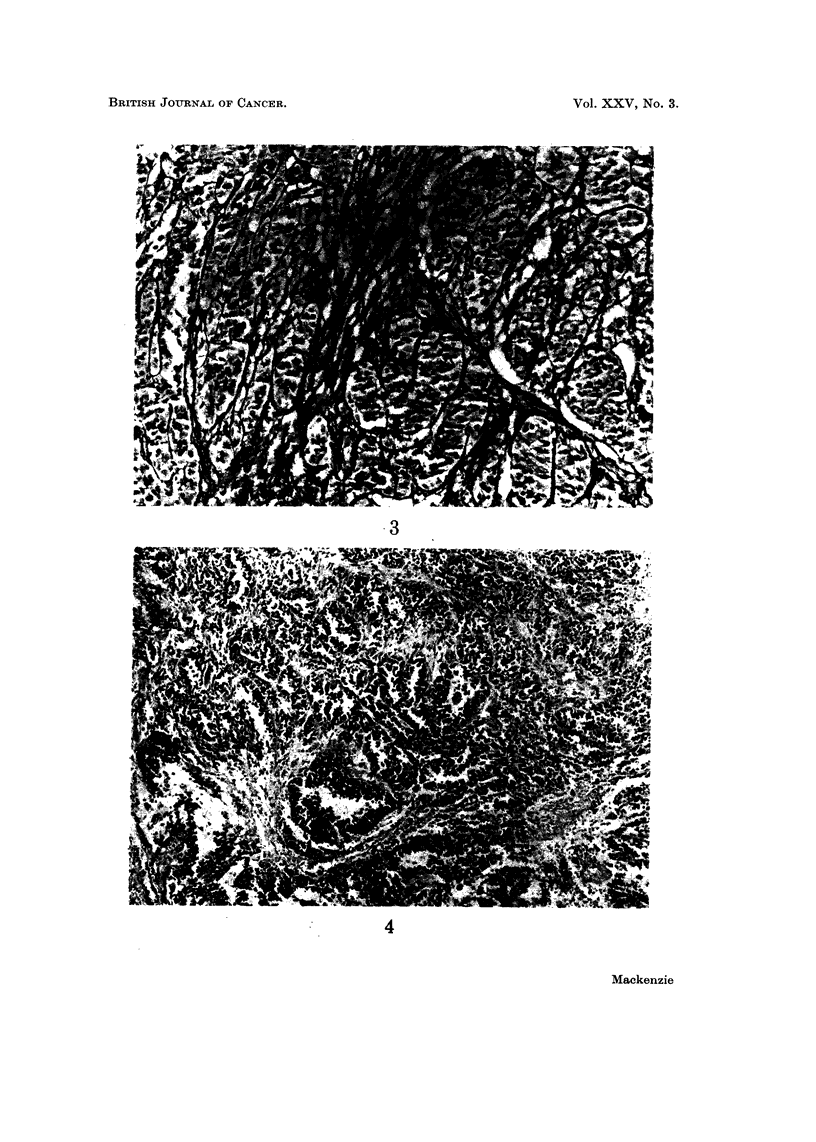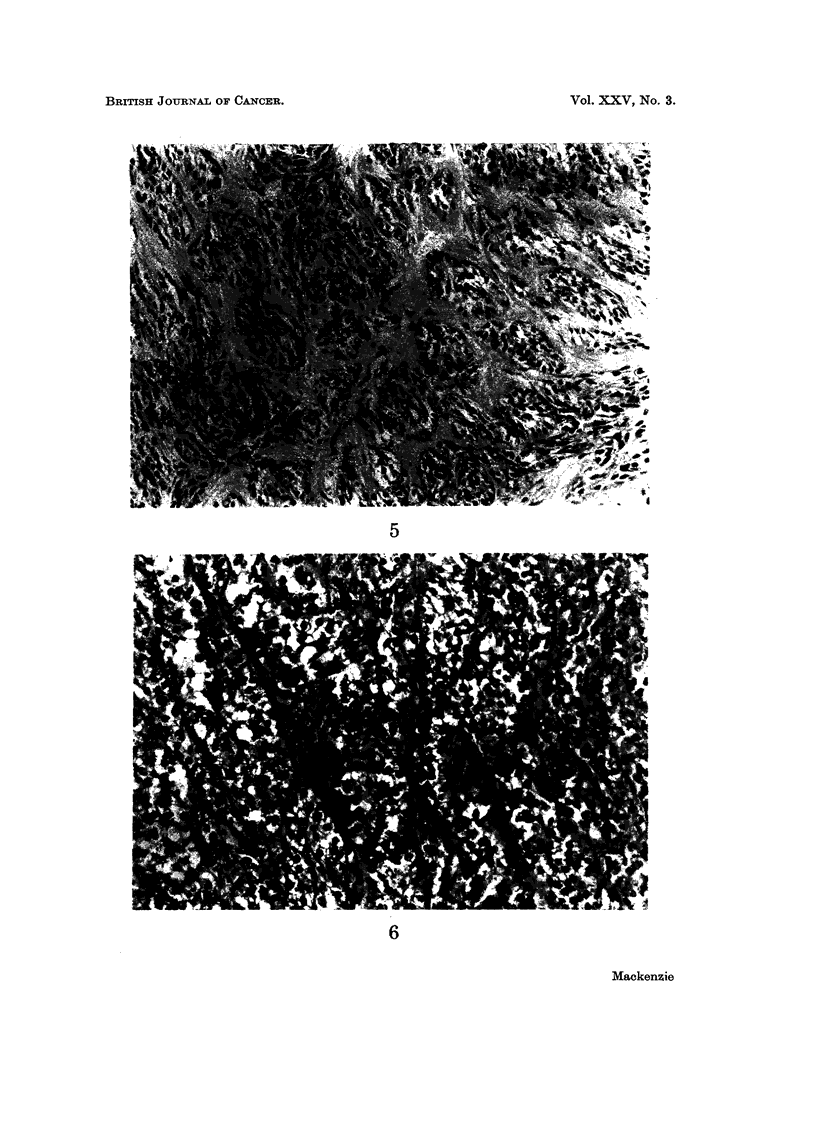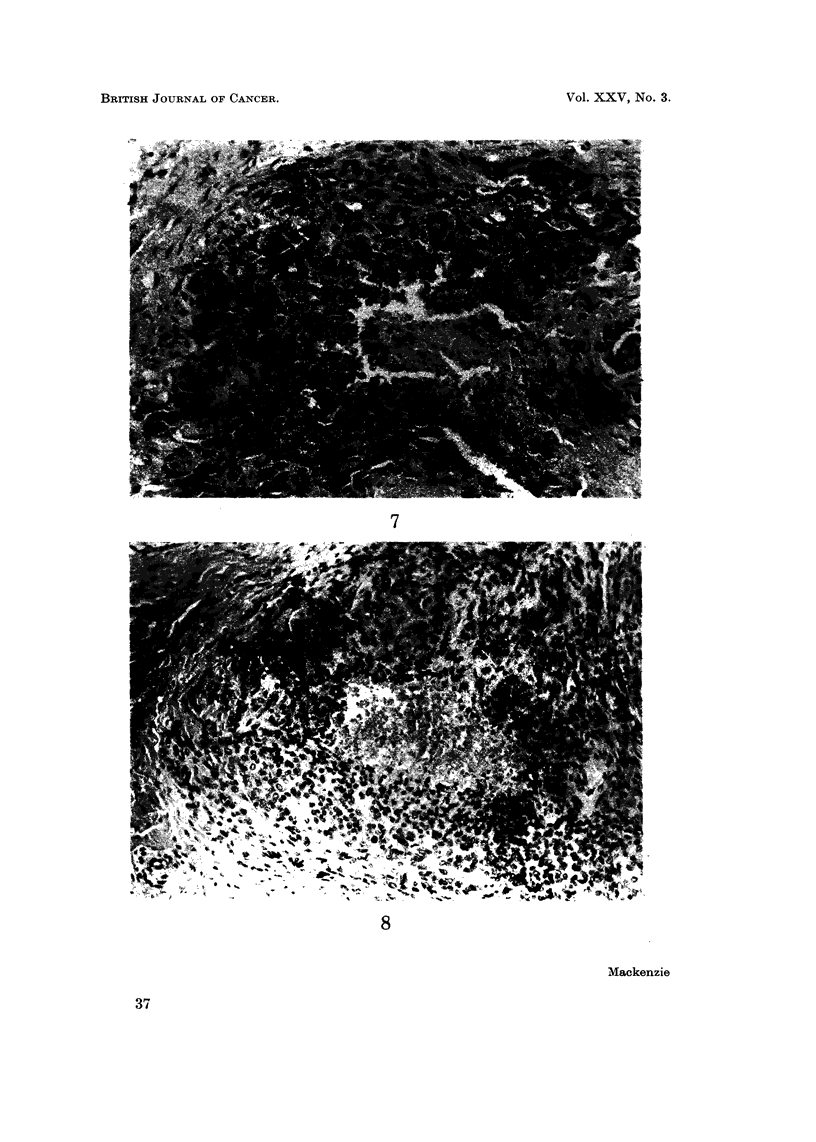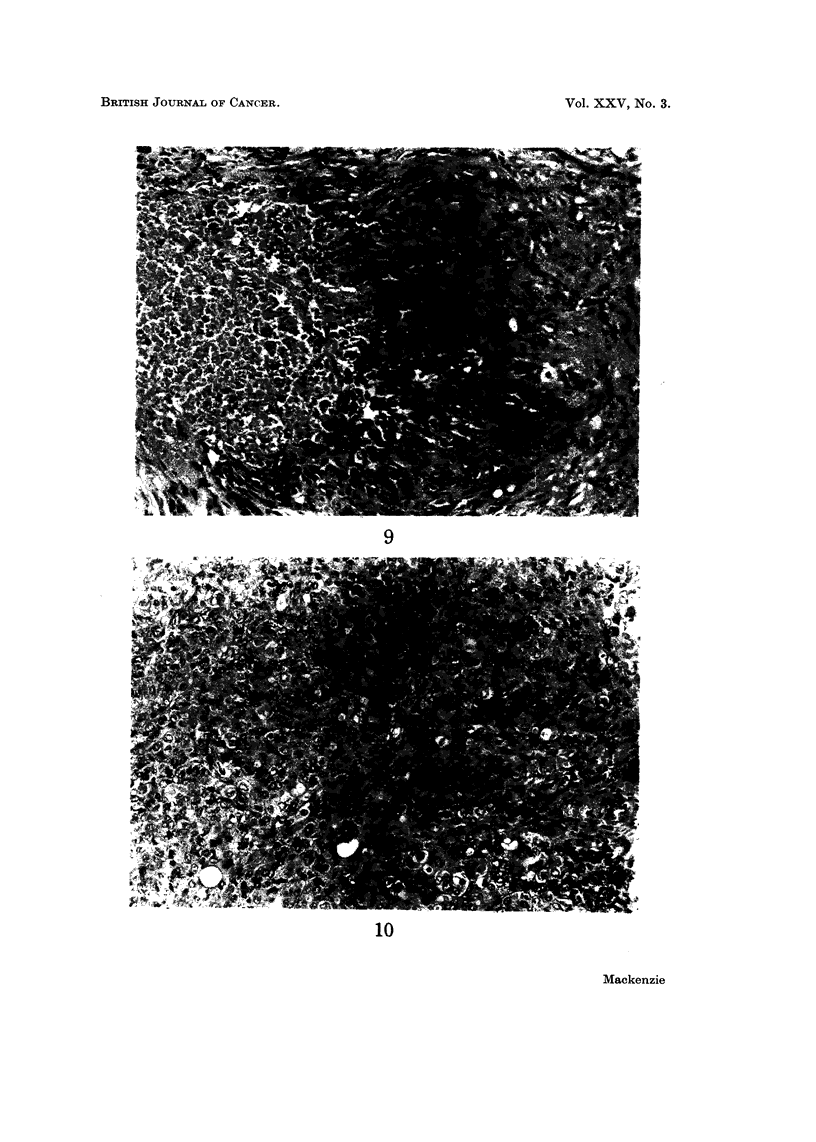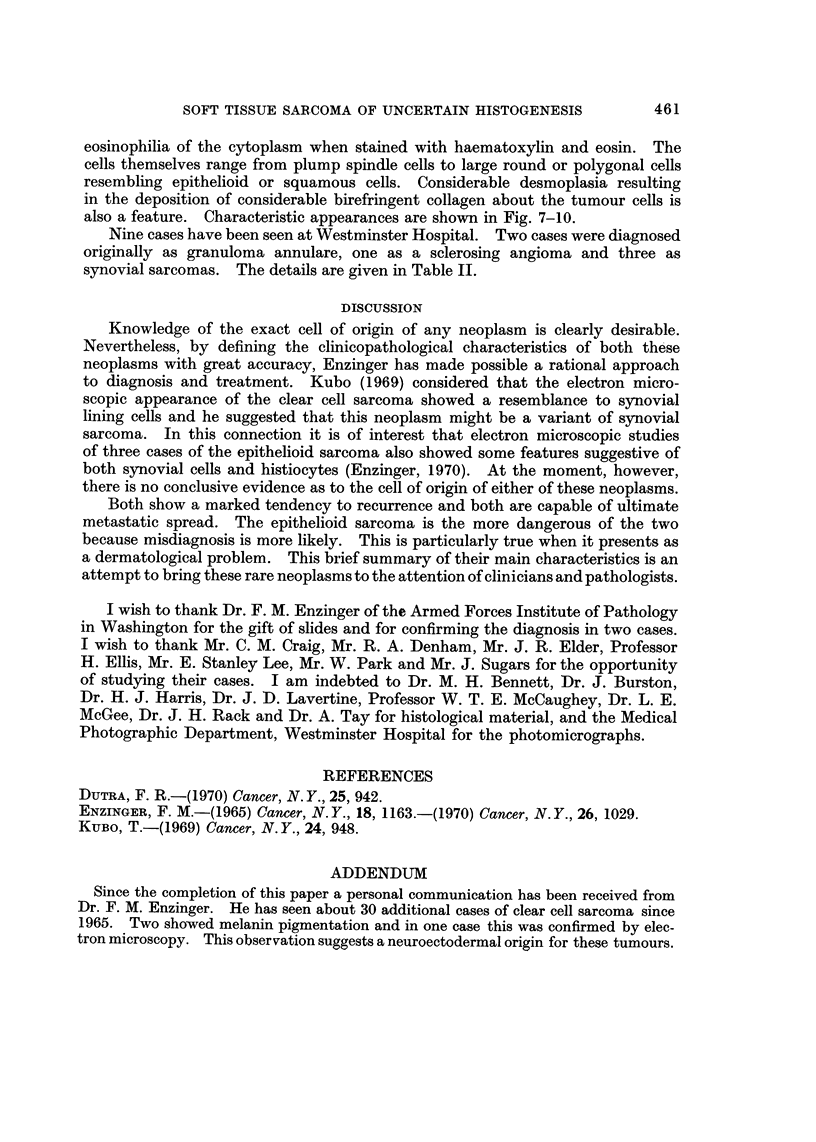# Two Types of Soft Tissue Sarcoma of Uncertain Histogenesis

**DOI:** 10.1038/bjc.1971.58

**Published:** 1971-09

**Authors:** D. H. Mackenzie

## Abstract

**Images:**


### TWO TYPES OF SOFT TISSUE SARCOMA OF UNCERTAIN HISTOGENESIS

D. H. MACKENZIE

*From the Department of Morbid Anatomy, Westminster Hospital, London*

Received for publication July 30, 1971

**SUMMARY.— A brief account is given of two types of rare soft tissue sarcoma of uncertain histogenesis. The danger of mistaking the epitheloid sarcoma for a benign lesion is emphasized.**

ENZINGER (1965, 1970) delineated two rare types of sarcoma arising in soft tissues. Although the cell of origin is unknown in both types, these neoplasms have a characteristic histological appearance and natural history. The clear cell sarcoma of tendon and aponeurosis is unlikely to be mistaken for a benign tumour, but the epithelioid sarcoma is a sinister entity liable to be misdiagnosed as a benign granulomatous proliferation or, alternatively, as a carcinoma. There may seem little justification for recording a very few further cases, but in the last 9 months I have seen three cases of the epithelioid sarcoma all diagnosed as benign. In two of these the diagnosis was granuloma annulare. Therefore, a brief report of these cases in an English Journal and of several others found in the files of Westminster Hospital seemed justified. For comprehensive accounts readers are referred to Enzinger's definitive papers on the subject.

### *Clear Cell Sarcoma of Tendons and Aponeuroses*

Enzinger (1965) described 21 cases of this rare and unusual neoplasm. Kubo (1969) described the electron microscopic appearances of a case arising from the patellar tendon. Dutra (1970) recorded three further cases. In his Cases 1 and 2 there were considerable differences of opinion regarding the nature of the neoplasms and the photomicrographs are not entirely convincing. These tumours, which can occur at any age, usually present as a slowly growing painless mass. They are slightly more common in women. They arise from tendinous structures in the extremities and are commonest in the foot and ankle region. They pursue a slow but relentless clinical course with a tendency towards repeated recurrences and eventual metastases. In Enzinger's series 14 cases died with metastases particularly in the lungs and lymph nodes.

### *Pathology*

The tumours are usually firm, roughly spherical, with a smooth, nodular or coarsely lobulated surface. A pseudocapsule may be present. They are intimately associated with a tendon or aponeurosis. The size usually ranges between 2 cm. and 6 cm. They have a grey, white or matted cut surface and small cysts may be seen in a minority of cases.

TABLE I.—*Clear Cell Sarcomas*

| Case | Age | Sex | Site | Recurrences | Metastases | Treatment | Follow-up | Length of initial history | Initial diagnosis |
|---|---|---|---|---|---|---|---|---|---|
| 1 | 27 | M | Ankle | 0 | Inguinal nodes, lungs, pleurae, ribs | Excision, radiotherapy, later amputation | Died after 6½ years | Not known | Synovial sarcoma |
| 2 | 51 | M | Ankle | 0 | Multiple metastases. No P.M. | Excision, radiotherapy | Died after 3 years | 1 year | Synovial sarcoma |
| 3 | 19 | M | Ankle | 2 | Inguinal nodes, lung | Excision, radiotherapy | Alive with metastases 22 years | 6 months | Synovial sarcoma |
| 4 | 30 | M | Ankle | 0 | Popliteal fossa | Excision followed by amputation | Alive and well 6 years | Not known | Clear cell sarcoma |
| 5 | 40 | F | Thigh | — | Lost to follow up | | — | — | Clear cell sarcoma |
| 6 | 19 | F | Lower back | 0 | Inguinal nodes | Excision | Alive with metastases | 3½ years | Clear cell sarcoma |

TABLE II.—*Epithelioid Sarcoma*

| Case | Age | Sex | Site | Recurrences | Metastases | Treatment | Follow-up | Length of initial history | Initial diagnosis |
|---|---|---|---|---|---|---|---|---|---|
| 1 | 21 | F | Upper arm | 0 | Lungs | Excision and radiotherapy | Died after 1 year | 7 months | Synovial sarcoma |
| 2 | 29 | M | Hand | 1 | — | Excision and later radiotherapy | Alive and free of disease 15 years | 2 years | Synovial sarcoma |
| 3 | 24 | F | Hand | 1 | — | Excision and radiotherapy | Alive and free of disease 10 years | Not known | Synovial sarcoma |
| 4 | 42 | F | Finger (tip) | 1 | Nodule at base of same finger | Excision and later amputation of finger | Alive and free of disease 3 years | 1 year | Granuloma annulare |
| 5 | 23 | M | Forearm | 1 | Skin, lungs | Excision and radiotherapy and amputation | Alive with metastases 4 years | 3 years | Sclerosing angioma |
| 6 | 18 | M | Finger | 1 | — | Excision | Alive and well 1 year | Not known | Granuloma annulare |
| 7 | 63 | F | Near knee | 1 | Lymph nodes | Excision and radiotherapy | Alive and free of disease 1½ years | Not known | Epithelioid sarcoma |
| 8 | 35 | F | Forearm | — | A recent case | — | — | — | Epithelioid sarcoma |
| 9 | 2½ | F | Hand | 1 | A recent case | — | — | — | Epithelioid sarcoma |

Histologically the tumours are made up of nests and fascicles of pale fusiform cells of epithelioid appearance which often have prominent nucleoli. Connective tissue septa often enclose these cellular aggregates. Multi-nucleated giant cells have been described in over half the cases. The characteristic picture is shown in Fig. 1–6. In one of my own cases the histological picture suddenly changed to a pattern markedly reminiscent of an alveolar rhabdomyosarcoma (Fig. 4). No cross striations were identified and Enzinger who was kind enough to examine the sections regarded the change as being due to " tissue shrinkage, compounded by the effect of muscle infiltration and fibrosis " (personal communication, 1970).

In his experience the typical histological picture may be lost in recurrent or metastatic lesions. Diagnoses which have been applied to this neoplasm in the past include synovial sarcoma, fibrosarcoma, alveolar soft part sarcoma and malignant melanoma. A detailed discussion is given in Enzinger's original paper.

I have had the opportunity of studying six cases. The details of these are shown in Table I.

### *Epithelioid Sarcoma*

Enzinger (1970) gave a comprehensive account of this unusual neoplasm and his findings are summarized briefly.

One of the most remarkable things about this neoplasm has been the multiplicity of diagnoses applied to it. Enzinger lists 11 different benign diagnoses to which must be added granuloma annulare and 20 different malignant ones. The most frequent diagnoses were granuloma and synovial sarcoma.

These tumours can occur at any age but 70% occur between 10 and 34 years. The male/female ratio is approximately 3 : 1. They tend to arise as firm subcutaneous nodules or as chronic ulcers of the skin. Pain is a variable feature. In Enzinger's series of 62 cases all occurred in the extremities except two which arose in the scalp. The commonest site was the volar aspect of the fingers. The tendency was towards recurrences and ultimate metastatic spread. Follow-up information on 54 patients (87%) revealed slow, relentless clinical course with frequent recurrence (85%) and late metastasis (30%). The lungs and the scalp were the commonest site for metastases.

Histologically these tumours are characterised by the nodular arrangement of the tumour cells, their tendency to undergo necrosis and in many cases by the

---

EXPLANATION OF PLATES

FIG. 1.—Case 1. Clear cell sarcoma. Showing nests and fascicles of pale fusiform cells. H. and E. × 145.

FIG. 2.—Case 1. Clear cell sarcoma. Showing cells with pale cytoplasm and well defined nucleoli. H. and E. × 410.

FIG. 3.—Case 1. Clear cell sarcoma. Bundles of cells outlined by reticulin. Gordon and Sweets. × 150.

FIG. 4.—Case 1. Clear cell sarcoma. Showing an appearance reminiscent of an alveolar rhabdomyosarcoma. H. and E. × 95.

FIG. 5.—Case 4. Clear cell sarcoma. A similar appearance to Fig. 1. H. and E. × 150.

FIG. 6 —Case 6. Clear cell sarcoma. Showing sheets of pale cells interspersed by thin fibrous trabeculae. H. and E. × 205.

FIG. 7 —Case 3. Epithelioid sarcoma. Showing epithelioid-like cells surrounding an area of necrosis. H. and E. × 165.

FIG. 8.—Epithelioid sarcoma. A similar picture. H. and E. × 145. (By courtesy of Dr. F. M. Enzinger.)

FIG. 9.—Case 5. Epithelioid sarcoma. A metastatic nodule modified by radiotherapy. H. and E. × 145.

FIG. 10.—Case 6. Epithelioid sarcoma. An area devoid of necrosis. H. and E. × 185.

1

2

Mackenzie

3

4

Mackenzie

5

6

Mackenzie

7

8

Mackenzie

9

10

Mackenzie eosinophilia of the cytoplasm when stained with haematoxylin and eosin. The cells themselves range from plump spindle cells to large round or polygonal cells resembling epithelioid or squamous cells. Considerable desmoplasia resulting in the deposition of considerable birefringent collagen about the tumour cells is also a feature. Characteristic appearances are shown in Fig. 7–10.

Nine cases have been seen at Westminster Hospital. Two cases were diagnosed originally as granuloma annulare, one as a sclerosing angioma and three as synovial sarcomas. The details are given in Table II.

### DISCUSSION

Knowledge of the exact cell of origin of any neoplasm is clearly desirable. Nevertheless, by defining the clinicopathological characteristics of both these neoplasms with great accuracy, Enzinger has made possible a rational approach to diagnosis and treatment. Kubo (1969) considered that the electron microscopic appearance of the clear cell sarcoma showed a resemblance to synovial lining cells and he suggested that this neoplasm might be a variant of synovial sarcoma. In this connection it is of interest that electron microscopic studies of three cases of the epithelioid sarcoma also showed some features suggestive of both synovial cells and histiocytes (Enzinger, 1970). At the moment, however, there is no conclusive evidence as to the cell of origin of either of these neoplasms.

Both show a marked tendency to recurrence and both are capable of ultimate metastatic spread. The epithelioid sarcoma is the more dangerous of the two because misdiagnosis is more likely. This is particularly true when it presents as a dermatological problem. This brief summary of their main characteristics is an attempt to bring these rare neoplasms to the attention of clinicians and pathologists.

I wish to thank Dr. F. M. Enzinger of the Armed Forces Institute of Pathology in Washington for the gift of slides and for confirming the diagnosis in two cases. I wish to thank Mr. C. M. Craig, Mr. R. A. Denham, Mr. J. R. Elder, Professor H. Ellis, Mr. E. Stanley Lee, Mr. W. Park and Mr. J. Sugars for the opportunity of studying their cases. I am indebted to Dr. M. H. Bennett, Dr. J. Burston, Dr. H. J. Harris, Dr. J. D. Lavertine, Professor W. T. E. McCaughey, Dr. L. E. McGee, Dr. J. H. Rack and Dr. A. Tay for histological material, and the Medical Photographic Department, Westminster Hospital for the photomicrographs.

### REFERENCES

Dutra, F. R.—(1970) *Cancer, N.Y.*, **25**, 942.
Enzinger, F. M.—(1965) *Cancer, N.Y.*, **18**, 1163.—(1970) *Cancer, N.Y.*, **26**, 1029.
Kubo, T.—(1969) *Cancer, N.Y.*, **24**, 948.

### ADDENDUM

Since the completion of this paper a personal communication has been received from Dr. F. M. Enzinger. He has seen about 30 additional cases of clear cell sarcoma since 1965. Two showed melanin pigmentation and in one case this was confirmed by electron microscopy. This observation suggests a neuroectodermal origin for these tumours.